# Successful Transition to Whole-Genome Sequencing and Bioinformatics to Identify Invasive *Streptococcus* spp. Drug Resistance, Alaska, USA

**DOI:** 10.3201/eid3113.241828

**Published:** 2025-05

**Authors:** Karen M. Miernyk, Sopio Chochua, Ben Metcalf, Alisa Reasonover, Brenna Simons-Petrusa

**Affiliations:** Centers for Disease Control and Prevention, Anchorage, Alaska, USA (K.M. Miernyk, A. Reasonover, B. Simons-Petrusa); Centers for Disease Control and Prevention, Atlanta, Georgia, USA (S. Chochua, B. Metcalf)

**Keywords:** *Streptococcus pneumoniae*, *Streptococcus agalactiae*, *Streptococcus pyogenes*, invasive *Streptococcus*, invasive pneumococcal disease, whole-genome sequencing, bioinformatics, antimicrobial resistance, bacteria, respiratory infections, streptococci, Alaska, United States

## Abstract

The Centers for Disease Control and Prevention’s Arctic Investigations Program evaluated whole-genome sequencing (WGS) workflows and bioinformatics pipelines developed by the Centers’ *Streptococcus* Laboratory. We compared WGS-based antimicrobial drug resistance predictions with phenotypic testing for group B (n = 130) and group A (n = 217) *Streptococcus* and *Streptococcus pneumoniae* (n = 293). Isolates were collected in Alaska during January 2019–February 2021. We also included a historical phenotypically nonsusceptible subset. Concordances between phenotypic testing and WGS predictions were 99.9% (895/896) for group B *Streptococcus*, 100% (1,298/1,298) for group A *Streptococcus*, and 99.98% (3,516/3,517) for *S. pneumoniae*. Common resistance determinants were *ermTR*, *ermB*, and *mef* for macrolides, *tetM* for tetracyclines, and g*yrA* and *parC* for levofloxacin. *S. pneumoniae* trimethoprim/sulfamethoxazole nonsusceptibility was associated with *folP* gene insertions and *folA* mutations. In 2022, the Arctic Investigations Program transitioned *Streptococcus* spp. workflows to WGS, enabling more rapid monitoring and prevention of invasive disease.

The Arctic Investigations Program (AIP), the Centers for Disease Control and Prevention (CDC) infectious disease field station in Alaska, USA, began surveillance of invasive pneumococcal disease (IPD) in 1986 (*1*) and added invasive cases of *Streptococcus agalactiae* (group B *Streptococcus*; GBS) and *S. pyogenes* (group A *Streptococcus*; GAS) in 2000 (*2*,*3*). Clinical laboratories across Alaska send case isolates to AIP for species confirmation and strain characterization. Data from AIP’s Invasive Bacterial Disease Surveillance (IBDS) are critical for understanding disease patterns. Alaska Native persons have higher rates of S*treptococcus* spp. infections than non–Alaska Native persons (*3*,*4*). In 2015, CDC’s Office of Advanced Molecular Detection, National Center for Emerging and Zoonotic Infectious Diseases, granted AIP funds to develop a technical and bioinformatics infrastructure and workforce to operationalize microbial genomics and enhance AIP’s ability to detect outbreaks, provide information about genetic lineage, and identify genetic determinants associated with antimicrobial drug resistance and virulence.

CDC’s *Streptococcus* Laboratory, Division of Bacterial Diseases, National Center for Immunization and Respiratory Diseases, transitioned its Active Bacterial Core Surveillance workflows to whole-genome sequencing (WGS) in 2015 (*5*–*7*). WGS analyses enable antimicrobial drug susceptibility predictions and strain typing, establish mechanisms of resistance, identify genotypes, and characterize surface protein antigens without requiring the labor and specialized skills needed for conventional phenotypic characterization. WGS also contributes to outbreak and disease transmission investigations (*8*–*10*). After a 2016–2017 GAS *emm* type 26.3 outbreak investigation (*8*), AIP began discussing WGS technology transfer with the *Streptococcus* Laboratory. We describe WGS validation, antimicrobial susceptibility mechanisms, and next steps for *Streptococcus* spp. WGS at AIP. This work was reviewed by a CDC research review board, which deemed it not research.

## Materials and Methods

We validated isolates collected during 2019 and during January–February 2021. We also included a subset of isolates from 1986–2018 (*S. pneumoniae*) and 2000–2018 (GAS and GBS) because phenotypic testing showed those isolates were nonsusceptible to >1 antimicrobial drug. AIP had determined antimicrobial drug MICs, *S. pneumoniae* serotypes, and GAS *emm* types previously by using phenotypic microbiologic methods. We cultured isolates according to a previously established protocol (Appendix). We extracted genomic DNA and prepared DNA libraries by using Nextera DNA Flex with 96 dual indices (Illumina, https://www.illumina.com). We pooled libraries and performed WGS by using a MiSeq instrument and MiSeq v2 500 cycle reagent kit (Illumina). We used bioinformatic pipelines developed and validated by the CDC *Streptococcus* Laboratory (https://github.com/BenJamesMetcalf) (Appendix) (*5*–*7*).

We compared MICs from phenotypic testing with MICs predicted by WGS. We considered results to be preliminarily discordant when MICs between phenotypic and WGS methods differed by >2 dilutions. We retested isolates with discordant results by using Etest (bioMérieux, https://www.biomerieux.com), BD BBL Sensi-Disc for the D-Zone (BD, https://www.bd.com), or other disk diffusion methods (BD). We confirmed results were discordant when the original phenotypic result agreed with follow-up testing but continued to differ from the WGS prediction by >2 dilutions.

For GAS isolates, we compared *emm* type results from Sanger sequencing with WGS assignments. We extracted DNA again from isolates that had preliminary discordant results and tested all 3 extracts (original extract, extract for WGS, and reextraction) by using Sanger sequencing. We confirmed those results were discordant if the Sanger sequencing results continued to differ from the WGS assignment.

For *S. pneumoniae* isolates, we compared phenotypic serotype results with WGS assignments. We tested those isolates with preliminary discordant results by using the Immulex Pneumotest (SSI Diagnostica A/S, https://ssidiagnostica.com) with Quellung reaction confirmation. We confirmed results were discordant when the follow-up testing agreed with the original serotype result and continued to differ from the WGS assignment.

## Results

We tested 130 GBS (82 from 2019/2021, 48 historical), 217 GAS (169 from 2019/2021, 48 historical), and 293 *S. pneumoniae* (203 from 2019/2021, 90 historical) isolates. Initial comparisons showed a preliminary concordance of 99.4% (891/896) for GBS, 99.2% (1,288/1,298) for GAS, and 98.7% (3,470/3,517) for *S. pneumoniae*. Follow-up testing confirmed the WGS prediction for 4/5 (80%) GBS isolates, 10/10 (100%) GAS isolates, and 46/47 (97.9%) *S. pneumoniae* isolates. Final concordance was 99.9% (895/896) for GBS, 100% (1,298/1,298) for GAS, and 99.98% (3,516/3,517) for *S. pneumoniae*.

Initial phenotypic analysis showed 192 nonsusceptible results for GBS isolates (Table 1). Two GBS isolates exhibiting high (MIC ≥8 μg/mL) levofloxacin resistance contained amino acid substitutions in the GyrA subunit of DNA gyrase (S81L) and the ParC subunit of topoisomerase IV, of which 1 had S79F and 1 had S79Y. Tetracycline nonsusceptibility was most often caused by the *tetM* determinant (n = 37); the *tetO* determinant accounted for the other 7 instances. The 5 preliminary discordant results for GBS occurred with erythromycin (n = 2) and clindamycin (n = 3) (Table 2); follow-up testing confirmed the WGS prediction for 4/5 (80%) isolates. Most combined erythromycin and clindamycin nonsusceptibility was associated with the presence of the 23S rRNA methylase genes, *ermTR* (n = 42) or *ermB* (n = 19). All *ermB*-positive isolates were constitutively clindamycin resistant, whereas 12/42 (28.6%) *ermTR*-positive isolates were inducibly clindamycin resistant. Erythromycin resistance and clindamycin susceptibility (M phenotype) were detected in 13 isolates with the *mef*-positive genotype. Two isolates contained the *lsaC* gene, which confers resistance to clindamycin. One GBS isolate with a discordant result contained the *lsaC* gene but was phenotypically sensitive to clindamycin. Three isolates constitutively resistant to erythromycin and clindamycin contained multiple resistance mechanisms, 1 each of *ermB* plus *lsaC*, *ermB* plus *mef*, and *lsaC* plus *mef*.

**Table 1:** Initial phenotypic testing results for isolates included in whole-genome sequencing workflow validation to identify invasive *Streptococcus* spp. drug resistance in Alaska, USA, during 2000–2021*

Antimicrobial drug	*Streptococcus agalactiae* isolates				*S. pyogenes* isolates		
	Total no.	No. susceptible	No. nonsusceptible		Total no.	No. susceptible	No. nonsusceptible
Ampicillin	112	112	0		165	165	0
Cefotaxime	45	45	0		34	34	0
Clindamycin	118	51	67		180	108	72
Erythromycin	119	40	79		180	99	81
Levofloxacin	118	116	2		181	177	4
Linezolid	103	103	0		176	176	0
Penicillin	115	115	0		166	166	0
Tetracycline	47	3	44		35	2	33
Vancomycin	119	119	0		181	181	0

*130 *S. agalactiae* and 217 *S. pyogenes* isolates were analyzed.

**Table 2:** Discordance between phenotypic testing and whole-genome sequence predictions along with resistance determinants for *Streptococcus* spp. in study to identify invasive *Streptococcus* spp. drug resistance, Alaska, USA, 1986–2021*

Organism resistance mechanism	Antimicrobial drug	WGS predicted MIC, μg/mL	Initial phenotypic MIC, μg/mL	Follow-up testing for >2 dilution discrepancy		
				MIC, μg/mL	Agrees with WGS, no.	True discrepancy, no.
*Streptococcus agalactiae*						
*lsaC* gene	Clindamycin	>1, R	0.25, S	0.25, S	0	1
negative	Clindamycin	<0.25, S	2, R	0.064, S	1	0
	Clindamycin	<0.25, S	2, R	0.047, S	1	0
	Erythromycin	<0.25, S	0.5, I	0.047, S	1	0
	Erythromycin	<0.25, S	8, R	0.064, S	1	0
*S. pyogenes*						
*ermTR* gene	Clindamycin†	>1, R	<0.12, S	All D-Zone +	8	0
Negative	Tetracycline	<2, S	4, I	0.125, S	1	0
	Erythromycin	<0.25, S	2, R	0.064, S	1	0
*S. pneumoniae*						
*mef* gene	Erythromycin	8, R	0.12, S	4, R	1	0
negative	Chloramphenicol	<2, S	8, R	2, S	1	0
	Levofloxacin	<2, S	4, I	0.5, S	1	0
	Erythromycin	0.06, S	1, R	0.06, S	1	0
	Tetracycline	<0.25, S	4, I	0.125, S	1	0
	Tetracycline	<0.25, S	>16, R	0.25, S	1	0
	Tetracycline	<0.25, S	>16, R	0.125, S	1	0
	Quinupristin, dalfopristin	<1, S	>4, R	Sensitive‡	13	0
	Rifampin	<1, S	>4, R	<0.064, S	24	0

*I, intermediate resistance; R, resistant; S, susceptible; WGS, whole-genome sequencing; +, positive. †Follow-up testing for clindamycin used BD BBL Sensi-Disc for the D-Zone test (BD, https://www.bd.com). ‡Determined by disk diffusion.

Initial phenotypic analysis showed 190 nonsusceptible results for GAS isolates (Table 1). Three isolates with the S81F substitution in ParC had intermediate levofloxacin resistance; 1 isolate with high (MIC ≥8 μg/mL) levofloxacin resistance contained amino acid substitutions in both GyrA (S81Y) and ParC (D85N) protein subunits. We identified preliminary discordant GAS results when comparing tetracycline (n = 1), clindamycin (n = 8), and erythromycin (n = 1) (Table 2); follow-up testing confirmed the WGS predictions for all 10 of those isolates. All 32 isolates nonsusceptible to tetracycline contained the *tetM* determinant. Most combined erythromycin and clindamycin nonsusceptibility was associated with the presence of *ermTR* (n = 71), *ermB* (n = 5), or *ermT* (n = 1) gene determinants. All *ermB*-positive and *ermT*-positive isolates were constitutively clindamycin resistant, whereas 29/71 (40.8%) *ermTR*-positive isolates were inducibly clindamycin resistant. Three isolates constitutively resistant to erythromycin and clindamycin contained >1 resistance mechanism, *ermT* plus *ermTR* (n = 2) and *ermTR* plus *lsaC* (n = 1).

We completed *emm* typing by Sanger sequencing for 201 GAS isolates, identifying 37 *emm* types. The initial comparison between Sanger sequencing and WGS showed 199/201 (99.0%) *emm* type concordance. Sanger sequencing of a third DNA extraction from both isolates confirmed 100% WGS concordance.

Initial phenotypic analysis showed 467 nonsusceptible results for *S. pneumoniae* (Table 3). At initial comparison, 13 isolates were nonsusceptible to quinupristin/dalfopristin and 24 isolates were nonsusceptible to rifampin; follow-up testing confirmed the WGS prediction for all 37 isolates (Table 2). One isolate exhibited high (MIC ≥8 μg/mL) fluoroquinolone resistance and had amino acid substitutions in both GyrA (S81F) and ParC (S79F) subunits. All 15 isolates containing the chloramphenicol acetyl transferase (*cat*) gene were resistant to chloramphenicol, and all 35 isolates nonsusceptible to tetracycline contained the *tetM* determinant. Three isolates without a WGS-identified tetracycline resistance mechanism were phenotypically nonsusceptible to tetracycline at initial testing; follow-up testing confirmed the WGS prediction for all 3 isolates.

**Table 3:** Initial phenotypic testing results for 293 *Streptococcus pneumoniae* isolates used for whole-genome sequencing workflow validation in Alaska in study to identify invasive *Streptococcus* spp. drug resistance, 1986–2021

Antimicrobial drugs	Total no. isolates	No. susceptible isolates	No. nonsusceptible isolates
Cefotaxime	75	38*	37*
Ceftriaxone	278	271*	26*
Chloramphenicol	293	278	15
Clindamycin	274	248	26
Erythromycin	291	221	70
Levofloxacin	277	276	1
Linezolid	271	271	0
Meropenem	278	247	31
Penicillin	293	218*	75*
Rifampin	246	222	24
Quinupristin/dalfopristin	275	262	13
Tetracycline	87	49	38
Trimethoprim/sulfamethoxazole	286	175	111
Vancomycin	293	293	0

*Determined by using the Clinical and Laboratory Standards Institute (https://www.clsi.org) breakpoint for meningitis cases.

We detected nonsusceptibility to trimethoprim/sulfamethoxazole in 111 *S. pneumoniae* isolates; 62 of those had an insertion of 1 or 2 codons within the *folP* gene, conferring intermediate drug resistance. The remaining 49 isolates were double mutants, leading to full resistance consisting of a *folA* gene mutation (I100L amino acid substitution) and insertion of 1 or 2 codons within *folP*. Most combined erythromycin and clindamycin nonsusceptibility was associated with the presence of *ermB* (n = 16); all of those were constitutively clindamycin resistant. An additional 3 isolates were *ermB* positive, phenotypically erythromycin nonsusceptible, but were not tested phenotypically for clindamycin nonsusceptibility. Ten isolates constitutively resistant to erythromycin and clindamycin were *ermB* and *mef* positive; 41 isolates with the M phenotype were *mef* positive. Two isolates were initially discordant for erythromycin sensitivity; 1 was *mef* positive but phenotypically sensitive, and 1 did not contain a resistance mechanism but was phenotypically nonsusceptible. Follow-up testing confirmed the WGS predication for both isolates.

Most *S. pneumoniae* isolates had MIC predictions related to penicillin-binding protein (PBP) gene types that indicated sensitivity to ceftriaxone (n = 271), meropenem (n = 247), penicillin (n = 218), and cefotaxime (n = 38) (Table 3). Penicillin and cefotaxime MIC predictions were concordant with phenotypic testing for all *S. pneumoniae* isolates. One initial discordant result for meropenem and 3 initial discordant results for ceftriaxone were observed; all 4 isolates were phenotypically sensitive, but WGS predicted nonsusceptibility (Table 4). Follow-up testing confirmed the WGS prediction for 3/4 (75%) isolates; 1 isolate was predicted to be nonsusceptible to ceftriaxone but was sensitive according to both the initial and additional phenotypic testing.

**Table 4:** Discordant *Streptococcus pneumoniae* β-lactam resistance predicted by penicillin-binding protein sequences compared with phenotypic testing results, Alaska, 1986–2021*

Antimicrobial drug	WGS predicted MIC, μg/mL	Initial phenotypic MIC, μg/mL	Follow-up testing for ≥2 dilution discrepancy		
			MIC, μg/mL	Agrees with WGS, no.	True discrepancy, no.
Meropenem	1, R	0.25, S	1, R	1	0
Ceftriaxone†	1, I/S	<0.5, S/S	1, I/S	2	0
	1, I/S	<0.5, S/S	0.019, S/S	0	1

*I, intermediate resistance; R, resistant; S, susceptible; WGS, whole-genome sequencing. †Determined by using the Clinical and Laboratory Standards Institute (https://www.clsi.org) breakpoints for meningitis/nonmeningitis cases.

Quellung serotyping was completed for 258 *S. pneumoniae* isolates, which comprised 30 serotypes. During the initial comparison, 2 discordant results were observed, likely related to an isolate mixup during phenotypic testing; additional testing confirmed the WGS-assigned result for both, indicating 100% concordance.

## Discussion

Despite data published by the CDC’s *Streptococcus* Laboratory supporting the accuracy of WGS-based antimicrobial drug susceptibility predictions (*5*–*7*), some collaborators have not pursued WGS workflows because they are uncertain those predictions are accurate (K.M. Miernyk, unpub. data). The antimicrobial susceptibility data shown here provide further evidence to address those concerns. For the 3 *Streptococcus* spp., we found 99.96% (5,709/5,711) concordance between phenotypic testing and genomic predictions. In addition, the WGS predictions were more accurate than phenotypic testing. Of 62 initial discordant results, WGS was confirmed to be correct for 60 (97%) of those.

Antimicrobial drug susceptibility data are needed to inform patient treatment and develop population treatment guidelines. IPD disproportionately impacts Alaska Native persons. AIP’s IBDS indicated the IPD rates associated with serotypes not targeted by the licensed 13-valent pneumococcal conjugate vaccine (PCV13) were 27.2/100,000 Alaska Native persons and 6.7/100,000 non–Alaska Native persons during April 2010–December 2013 (*4*). Similarly, the percentage of persons carrying non-PCV13 serotype *S. pneumoniae* in their nasopharynx that was nonsusceptible to erythromycin or penicillin increased significantly from 16.8% (n = 709) during 2008–2011 to 26.5% (n = 1,466) during 2012–2015 (*11*). This finding suggests Alaska Native persons might be at increased risk for IPD caused by *S. pneumoniae* that are resistant to commonly used antimicrobial drugs. Capsular *S. pneumoniae* serotyping data are necessary to evaluate vaccines for IPD prevention. Finally, GAS *emm* typing data predict the M protein serotype (*12*), a potential target for vaccines to prevent invasive GAS infections. Traditional phenotypic methods are time consuming and require many different specialized technical skills, reagents, and consumables. The WGS workflow described here provides those data for 48 samples simultaneously by using 1 method. In addition, as the COVID-19 pandemic revealed, supply chains can be unreliable. WGS provides data in a single workflow, enabling a more streamlined process with fewer consumables and reagents.

AIP has not previously had the capacity to consistently characterize resistance mechanisms in any *Streptococcus* spp. bacteria. To better elucidate changes in macrolide resistance after PCV13 introduction, we briefly used PCR to characterize *ermB* and *mef* macrolide resistance mechanisms in *S. pneumoniae* (*13*). However, we have not investigated those mechanisms in GAS or GBS collected from persons in Alaska, which could be of particular importance for GAS. Candidate GAS vaccines target the M protein (*14*), and >275 known M types exist. Therefore, vaccine pressure on targeted M types could affect circulating strains of GAS, which has been observed for *S. pneumoniae* after PCV13 introduction (*15*). Macrolide nonsusceptibility is not uncommon for GAS, and it will be critical to understand whether changes in nonsusceptibility after vaccine introduction are caused by expansion of existing strains, by introduction of new strains, or by some other mechanism.

In conclusion, our antimicrobial susceptibility, serotype, and *emm* type validation data confirm the accuracy of WGS-based predictions for GAS, GBS, and *S. pneumoniae* when performed at AIP. The single WGS workflow is more efficient than multiple workflows needed for phenotypic testing. WGS pipelines identify previously unknown genotypic mechanisms for nonsusceptibility of *Streptococcus* spp. isolates collected in Alaska and provide additional data, such as GBS serotypes and multilocus sequence types. WGS also provides a genetic sequence for every isolate, which is available for future investigations. In 2022, AIP transitioned all IBDS workflows for *Streptococcus* spp. to WGS, and we continue to improve those processes. We have decreased cost by sequencing more extracts on a flow cell and have been increasing local analysis capabilities and data storage for more rapid and local pathogen detection. We also perform biannual phenotypic testing on a subset of isolates to monitor for new resistance genes. Future work includes validating a WGS workflow for GAS that can be used in remote field settings, enabling AIP to provide more rapid outbreak response.

Appendix Additional information for successful transition to whole-genome sequencing and bioinformatics to identify invasive *Streptococcus* spp. drug resistance, Alaska, USA.
