# Supplementary material for: Successful Transition to Whole-Genome Sequencing and Bioinformatics to Identify Invasive Streptococcus spp. Drug Resistance, Alaska, USA
Source: Emerg Infect Dis. 2025 May;31(Suppl 1):S103–8. doi: 10.3201/eid3113.241828 (PMC12078547; doi:10.3201/eid3113.241828)
Supplement: Appendix — Additional information for successful transition to whole-genome sequencing and bioinformatics to identify invasive Streptococcus spp. drug resistance, Alaska, USA. [file 24-1828-Techapp-s1.pdf]

Article DOI: https://doi.org/10.3201/eid3113.241828

EID cannot ensure accessibility for supplementary materials supplied by authors. Readers who have difficulty accessing supplementary content should contact the authors for assistance.

Successful Transition to Whole-Genome Sequencing and Bioinformatics to Identify Invasive *Streptococcus* spp. Drug Resistance, Alaska, USA

Appendix

Additional Materials and Methods

Initial phenotypic antimicrobial susceptibility testing was performed by using the microbroth dilution method. Interpretive categories and breakpoints were determined by using Table 2G (*Streptococcus pneumoniae*) and Table 2H-1 (Group A and B *Streptococcus*) from the Clinical and Laboratory Standards Institute M100, 29th edition, performance standards for antimicrobial susceptibility testing (*1*). We applied the meningitis breakpoints for antimicrobial drugs by using interpretive breakpoints that differed between meningitis and nonmeningitis cases.

Isolates were retrieved from the freezer, cultured onto trypticase soy agar with 5% sheep blood, and incubated overnight at 37°C with 5% CO_2. A single colony was subcultured onto the same medium, incubated again overnight, and then subcultured into 5 mL Todd Hewitt broth with 0.5% yeast extract. Genomic DNA was extracted with a modified QIAamp DNA Mini Kit protocol (QIAGEN, https://www.qiagen.com) that included prelysis with mutanolysin and lysozyme. DNA concentrations and quality were assessed by using an Invitrogen Qubit assay and Nanodrop 8000 spectrophotometer (both Thermo Fisher Scientific, https://www.thermofisher.com). Extracts with a concentration of <10 ng/µL or a 260:280 ratio of <1.8 were removed from the workflow; isolates were retrieved from the freezer and extracted again by using the same method. DNA libraries were prepared by using a Nextera DNA Flex library preparation kit with 96 dual indices (Illumina, https://www.illumina.com). Libraries were

quantified by using the Qubit assay as described and average peak size was determined by using an Agilent Bioanalyzer (Agilent Technologies, https://www/agilent.com). Forty-eight libraries were pooled, and WGS was performed by using a MiSeq instrument and MiSeq v2 500 cycle reagent kit (Illumina).

Bioinformatic pipelines developed and validated by the Centers for Disease Control and Prevention's *Streptococcus* Laboratory were used for analysis (*2–4*). Those pipelines obtain multilocus sequence type, serotype/*emm* type, pili, and drug resistance information from Illumina paired-end whole-genome sequences (*5*). The main program used to detect sequence types and document the presence of genes related to both serotype classification and antimicrobial drug resistance is the read mapping typing tool SRST2. Although read mapping worked well in detecting most loci used in our typing strategy, a different approach was needed when extracting potentially polymorphic mosaic regions (e.g., *folA*, *rplD*, and *S. pneumoniae* penicillin-binding protein [PBP] genes). This alternative strategy used the adaptor trimming tool Cutadapt version 1.6, the VelvetOptimiser version 2.2.5 assembler, the Prodigal version 2.60 gene predictor, and BLAST version 2.2.29 (https://blast.ncbi.nlm.nih.gov). WGS-based antimicrobial drug phenotype predictions were achieved, for the most part, through presence/absence typing of resistance determinants or by identifying specific mutations within those determinants. For β-lactam resistance, the Centers for Disease Control and Prevention provides a classification system in which a pneumococcal isolate is assigned to a PBP type according to sequence signatures in the transpeptidase domains of the 3 critical PBPs: PBP1a, PBP2b, and PBP2x. For *S. pneumoniae*, a random forest machine learning model trained on the amino acid at each position of the 3 transpeptidase domains was used to predict the $\log_2$ MIC (*6*).

References

1. Clinical and Laboratory Standards Institute. Performance standards for antimicrobial susceptibility testing; twenty-ninth edition (M100). Wayne (PA): The Institute; 2019.

2. . Chochua S, Metcalf BJ, Li Z, Rivers J, Mathis S, Jackson D, et al. Population and whole genome sequence based characterization of invasive group A streptococci recovered in the United States during 2015. mBio. 2017;8:e01422-17. PubMed https://doi.org/10.1128/mBio.01422-17

3. Metcalf BJ, Chochua S, Gertz RE Jr, Hawkins PA, Ricaldi J, Li Z, et al. Short-read whole genome sequencing for determination of antimicrobial resistance mechanisms and capsular serotypes of current invasive *Streptococcus agalactiae* recovered in the USA. Clin Microbiol Infect. 2017;23:574.e7–14. PubMed https://doi.org/10.1016/j.cmi.2017.02.021

4. Metcalf BJ, Chochua S, Gertz RE Jr., Li Z, Walker H, Tran T, et al. Using whole genome sequencing to identify resistance determinants and predict antimicrobial resistance phenotypes for year 2015 invasive pneumococcal disease isolates recovered in the United States. Clin Microbiol Infect. 2016;22:1002.e1–8. PubMed https://doi.org/10.1016/j.cmi.2016.08.001

5. Metcalf BJ, Gertz RE Jr., Gladstone RA, Walker H, Sherwood LK, Jackson D, et al. Strain features and distributions in pneumococci from children with invasive disease before and after 13-valent conjugate vaccine implementation in the USA. Clin Microbiol Infect. 2016;22:60.e9–29. PubMed https://doi.org/10.1016/j.cmi.2015.08.027

6. Li Y, Metcalf BJ, Chochua S, Li Z, Gertz RE Jr, Walker H, et al. Penicillin-binding protein transpeptidase signatures for tracking and predicting β-lactam resistance levels in *Streptococcus pneumoniae.* mBio. 2016;7:e00756-16. PubMed https://doi.org/10.1128/mBio.00756-16
